# Update on Zika Diagnostic Tests and WHO’s Related Activities

**DOI:** 10.1371/journal.pntd.0005269

**Published:** 2017-02-02

**Authors:** Arlene Chua, Irena Prat, Claudius Micha Nuebling, David Wood, Francis Moussy

**Affiliations:** 1 World Health Organization, Geneva, Switzerland; 2 Institute of Infectious Diseases and Epidemiology, Tan Tock Seng Hospital, Singapore; 3 Institute of Global Health, University of Geneva, Geneva, Switzerland; University of North Carolina at Chapel Hill, UNITED STATES

## Introduction

On 1 February 2016, WHO declared the clusters of microcephaly and neurological disorders and their potential link with Zika virus outbreaks in Brazil and French Polynesia to be a Public Health Emergency of International Concern (PHIC). Since then, there are 75 countries and territories reporting continued mosquito-borne transmission of Zika virus, with 69 countries experiencing a first outbreak of Zika virus since 2015.[1]

The current scientific evidence has now shown that infection with Zika virus in the prenatal period is associated with microcephaly and other serious brain abnormalities. [2] As of December 2016, microcephaly cases have been identified in 28 countries and territories, and Guillain–Barré syndrome cases have been reported in 20 countries and territories where Zika virus is circulating. [1]

There is currently a global scientific effort to gain a better understanding of the Zika virus, its vectors, modes of transmission, and the natural history of the disease. Key to this effort is having an accurate diagnosis of Zika virus infection; however, available tests are limited by several factors.

On 18 November 2016, the Director-General of WHO declared the end of the PHEIC based on the recommendations made by the Emergency Committee on Zika and microcephaly convened under the International Health Regulations (IHR).

WHO will continue its research and development (R&D) work on Zika under the WHO R&D Blueprint effort, which aims at reducing the time between declaration of a PHEIC and the availability of diagnostics tests, vaccines, antivirals, and other treatments that can save lives and avert a public health crisis. [3]

## Towards Better Diagnostic Tests—Target Product Profiles

In order to address the limitations and gaps of current diagnostic tests, WHO led the development of consensus-driven target product profiles (TPPs) with United Nations Children’s Fund (UNICEF), Pan American Health Organization (PAHO), and other organizations. The target product profiles define the desired characteristics of Zika diagnostic tests and are aspirational in nature. Several consultation meetings were held with key stakeholders in the public health and scientific community. This was followed by a public consultation from 22 March to 11 April 2016. Feedback from the consultation was incorporated into the final TPPs, which were published on 13 April 2016. [4]. Two TPPs were developed and are shown in Tables 1 and 2. The first TPP focuses on the detection of active infection with Zika virus and the second on evidence of prior infection. They are intended to meet the needs of different population groups and current Zika virus challenges. Some of the difficulties in the development of the TPP were due to the paucity of scientific evidence on Zika virus, including infection kinetics, viremia, and presence of virus in other body fluids as well as immune response, particularly in pregnant women. These difficulties, as well as additional considerations, are further discussed below and are important accompanying elements to interpret the TPPs.

**Table 1:** TPP for detection of active infection with ZIKV.

Intended Use	Diagnosis of Patients (Including Pregnant Women) with Active Infection		Blood Bank Testing	
Characteristic	Acceptable	Ideal	Acceptable	Ideal
Sampling and sample type	Whole blood from phlebotomy	Capillary blood or less invasive samples such as urine, saliva, others (if validated)	Plasma/serum	Same
Target level of health system and target user	Reference laboratory; trained laboratory technician	Point of care (primary health care clinic or higher); health care worker with minimal training	Blood collection facility or centralized blood banking facility/testing lab, results within 1–2 days for timely release of blood components	Same
Multiplexing	Single test for ZIKV	Simultaneous detection of pathogen-specific analytes for DENV, CHIKV^a^	Single test for ZIKV	Simultaneous detection of pathogens typically screened for blood bank testing^b^
Analytical sensitivity LoD	≤500 copies/mL^c^	In a multiplex test: 500 copies/mL^b^ in the presence of other target analytes, when other analytes are detected	<50 copies/mL	Same
Analytical specificity^d^	>98%	>99.5%	>99.5%	Same
Diagnostic sensitivity^e^	>95%	>98%	>95%	>98%

^a^ Analytes specific to other arboviruses (such as yellow fever) and other pathogens presenting with similar febrile syndromes may be added to the multiplex test as clinically and epidemiologically relevant to the setting of use.

^b^ Pooled or single unit testing needs to be assessed.

^c^ For nucleic acid tests.

^d^ No cross-reactivity with flaviviruses, alphaviruses, and other unrelated pathogens in laboratory (spiked samples) and in silico.

^e^ There is no current validated reference method to determine the clinical diagnostic sensitivity.

CHIKV, Chikungunya virus; DENV, Dengue virus; LOD, limit of detection; ZIKV, Zika virus.

**Table 2:** TPP for detection of evidence of prior infection to ZIKV.

Intended Use	Diagnosis of Prior Infection	
Characteristic	Acceptable	Ideal
Sampling and sample type	Whole blood from phlebotomy	Capillary blood or less invasive samples such as urine, saliva, others (if validated)
Target level of health system and target user	Reference laboratory; trained laboratory technician	Point of care (primary health care clinic or higher); health care worker with minimal training
Multiplexing	Single test for ZIKV	Simultaneous detection of previous infection with CHIKV and DENV, including DENV serotypes
Sensitivity	>95%	>98%
Specificity	>95%	>98%

CHIKV, Chikungunya virus; DENV, Dengue virus; ZIKV, Zika virus.

Current diagnosis of Zika virus infection is challenging for a variety of reasons. Zika virus infection is generally considered a mild infection, which it still is in the majority of cases. Approximately 80% of Zika infections are asymptomatic, and 20% of Zika virus infections present clinically with symptoms of mild fever, maculopapular rash, arthralgia, myalgia, conjunctivitis, and headache. [5] These symptoms overlap with infections caused by Dengue virus and Chikungunya virus, which are cocirculating in the affected countries. [6] Clinical presentation alone is not reliable to distinguish Zika virus from these other common causes of fever. Confirmation of Zika virus infection in patients presenting with <1 week of fever and consistent symptoms is based on detection of Zika virus RNA. Zika virus RNA levels in blood during the acute phase of infection are relatively low, with reported levels between 10^3^ and 10^5^ copies/mL. [7–9] Based on limited studies, Zika virus RNA in blood appears to be transient and can be detected from approximately 3 to 5 days after the onset of symptoms.[9,10] However, a case report of acute infection in a pregnant woman found that Zika virus RNA was detected in serum up to 10 weeks from the onset of symptoms. [11] Furthermore, Zika virus RNA has been detected in whole blood as late as 58 days after symptom onset without detection in text matrices in serum or plasma. [12] This finding may have implications for the design of diagnostic assays for detection of Zika virus RNA.

There are several small studies that suggest urine and saliva might be the preferred specimen type to diagnose active infection with Zika virus. [10,13,14] Zika virus RNA was detectable in urine for up to 20 days after its clearance from blood, with RNA levels in urine as high as 10^6^ copies/mL.[10]

Cross-reactivity with other flaviviruses is a significant concern and complicates interpretation as well as development of new assays. [8,9,15,16] This cross-reactivity is due to the high degree of structural and sequence homology between Zika and other flaviviruses. Using three-dimensional cryo-electron microscopy, Sirohi et al. showed that Zika virus shares the same structure as other flaviviruses. [17] A significant degree of sequence homology has also been demonstrated between Zika and other flaviviruses. A study comparing Zika virus with Dengue virus E proteins shows up to 53.9% identical amino acid sequence. [18] Available tests for Zika virus IgM cannot reliably distinguish between Zika virus and other flavivirus infection, such as Dengue virus, due to cross-reactivity. Until validated tests are available, positive tests for Zika virus IgM or Dengue virus IgM should be considered evidence for recent flavivirus infection but require additional confirmatory testing, using plaque-reduction neutralization test (PRNT) to differentiate between Dengue virus and Zika virus. PRNT is considered the most specific test to verify antibodies of closely related viruses. With the high volume of testing that is needed, however, this test is impractical given that it is performed by only a small number of laboratories worldwide, is labor-intensive and costly, and requires standardized reagents that may not be easily available. [19] Validated tests that reliably differentiate recent flavivirus infection from recent Zika virus infection are a priority need.

It is currently unknown if there is a difference in the immune response of pregnant women with Zika virus infection. The effect of pregnancy on antibody titers to other viruses has been described previously.[20,21] In a review of efficacy and duration of immunity with yellow fever vaccine, the immunogenicity in pregnant women varied depending on which trimester the vaccine was given.[22]

Available data on diagnosis of prenatal and antenatal Zika virus infection are derived from testing tissues of fetal losses, full-term infants who died shortly after birth, or infants with microcephaly. Pathologic evidence for Zika virus in the setting of microcephaly has been described in a number of case reports and small case series, with detection of Zika virus in brain tissue, amniotic fluid, and cerebrospinal fluid. [23–27] Isolation of Zika virus RNA in brain tissue from a fetus with microcephaly has been documented, with a high viral burden (6.5 x 10^7^ viral RNA copies per mg of tissue); in this report, Zika virus RNA was not detected in other tissue samples of the fetus. [24] Zika virus RNA was found in amniotic fluid, with an ELISA for anti-Zika virus IgM also found to be positive. [25] Adibi et al. describe data from an unpublished report, in which anti-Zika virus IgM, but not Zika virus RNA, was detected in the cerebrospinal fluid of 30 out of 31 babies born with microcephaly.[28]

The majority of stakeholders felt that multiplexing capability allowing simultaneous detection of several pathogens in a single sample—particularly Chikungunya virus and Dengue virus—is highly advantageous. Aside from Dengue virus and Chikungungya virus, the choice of pathogens in a multiplexed diagnostic should consider clinically consistent presentations, actionable information, and the needs of epidemiological surveillance. Not all pathogens will be relevant to all settings; nonetheless, the need to differentiate Chikungunya virus, Dengue virus, and Zika virus for both clinical and epidemiologic purposes is of immediate importance and is likely to remain relevant in the future.

Multiplexing using dual analytes in a single test (e.g., nucleic acid and immunoassay testing) [29] may also greatly improve the diagnosis of acute Zika virus infection. A test that could simultaneously detect both Zika virus RNA and anti-Zika virus IgM would cover the entire time period of acute Zika virus infection and may be particularly useful given the limited reliability of patient self-reports of the onset of fever and other symptoms.

Operational characteristics (such as specimen collection and processing requirements, storage requirements for reagents, laboratory logistics, etc.) should have attributes common to other ideal tests intended for public health use in low and middle income countries. [30–33]

## Acceptability of Testing and Ethical Considerations

While discussion on testing policy is not a standard component of a TPP, and because the greatest impact of Zika virus infection appears to occur during pregnancy, it is worthwhile to highlight the uncertainty surrounding Zika virus in pregnant women, the need for highly accurate Zika virus tests, and the need for a diagnostic testing algorithm(s) that can be applied during routine antenatal care or counseling. In a cohort of pregnant women in Brazil, 40% of the women who tested positive for Zika virus infection using the best available diagnostic tests in 2015–2016 declined imaging studies. Fear, related to the possible identification of fetal abnormalities related to Zika virus infection, was one of the reasons cited. [34] In this setting, the consequences of both false positive and false negative tests for Zika virus are uncertain but potentially profound. The TPPs defined here endorse clinical sensitivity and specificity values that will minimize, but not eliminate, false positive and false negative results.

Additional biologic, clinical, and social science research as well as consensus guidelines appropriate for different settings in which women receive antenatal care will be vital in establishing optimal testing strategies and algorithms for pregnant women.

## Validation of Available Zika Virus Tests

Commercial tests for Zika virus are becoming more available. There are at least seven nucleic acid detection lab-based tests (NAT), three serology tests that have received authorization for emergency use from regulatory agencies—Conformité Européene/in Vitro Diagnostic (CE/IVD), United States Food and Drug Administration Emergency Use Authorization (US FDA EUA), Agência Nacional de Vigilância Sanitária (ANVISA)—and several companies in various stages of product development.[35]

Following the declaration by the WHO Director-General of a PHEIC related to microcephaly cases potentially linked to Zika virus, the WHO Prequalification Team announced on 5 February 2016 that the Emergency Use Assessment and Listing (EUAL) procedure established during the 2014 Ebola virus disease outbreak was opened to candidate in vitro diagnostics (IVDs) intended for Zika virus diagnosis. The EUAL procedure was developed to expedite the availability of IVDs needed in public health emergency situations. It is intended to assist interested procurement agencies and Member States on the suitability for use of a specific IVD, based on a minimum set of available quality, safety, and performance data. The EUAL assessment includes a review of the product quality aspects, including manufacturing capability, an assessment of the studies supporting the performance of the test, and an independent performance evaluation complementing the manufacturer’s verification and validations studies.

EUAL submission requirements for manufacturers were set following a meeting of experts, including regulators from the affected region and the US FDA. The goal was to identify a set of minimal requirements that are aligned internationally. To date, WHO has received 33 applications for EUAL assessment from 22 manufacturers with applications for a range of technologies, including rapid test format antibody tests, NAT, immunofluorescence, and antigen detection tests.

A number of these applications have not met the WHO requirements for quality management systems, or have not undertaken sufficient verification and validation studies to comply with the EUAL requirements, and, as such, have withdrawn. Access to suitable specimens has been a major problem for manufacturers and has limited verification and validation studies. In addition, studies undertaken by manufacturers in Europe on infected travelers returning from the affected region may not provide sufficient challenge to the assay, as many of these patients have not previously been exposed to other flavivirus infections. This may limit the suitability of the assay in many of the Zika virus endemic countries. However, a number of the applications have evidence to indicate adequate performance and suitable manufacturing and are progressing through the assessment process. To date, two products—NAT assays—have passed the three-phase assessment process and were listed as eligible for WHO procurement.

## Zika Virus Reference Standards

In addition to the various molecular tests being developed for commercial use, there are also in-house developed assays and kits used for research purposes. [36] These laboratory-developed and commercial assays differ in the use of specimen, method, reagents, and instrumentation. Given the heterogeneity of these tests, it is important to have an internationally accepted reference preparation to compare and potentially standardize the different assays. WHO has established numerous reference preparations, most of them as WHO International Standards (WHO IS), for a number of different viral pathogens, including blood-borne viruses like HIV, hepatitis B virus, hepatitis C virus, further clinically relevant viruses, and, more recently, Ebola virus disease.

For Zika RNA, the biological standard for molecular tests had been characterized in an international study involving the majority of nucleic acid amplification technology (NAT)–based assays available.[37] The complete sequence of the Zika virus of this reference preparation has been published too. [38] This material was officially established as WHO IS by the respective WHO expert committee in October 2016; however, even prior to its establishment as WHO IS, the candidate material had been made available for test kit manufacturers, for regulatory authorities, and for users because of the urgent need.

The related work on WHO IS for serological assays detecting Zika antigen or anti-Zika antibodies is ongoing; unfortunately, the sourcing of suitable materials of human origin from Zika virus-affected regions is still an issue.

## Summary and Conclusions

There is an urgent need for new quality-assured diagnostics, particularly for decentralized use. WHO is facilitating the development, validation, and assessment of such diagnostics by issuing two consensus TPPs, by developing WHO reference preparations, and with its EUAL process.

Lack of access to well-characterized clinical samples of Zika virus continues to be a challenge. After the recent Ebola outbreak, WHO has developed a framework for developing a specimen library that will allow equitable access for researchers and assay developers. However, this has not yet been realized for this current outbreak.

While there has been some progress on the availability of Zika virus diagnostic tests, many challenges remain to meet the diagnostic needs of affected countries. Results of prospective studies will also be useful in designing the testing algorithm with the tests that will be available in the near term. There is a need to see beyond this current outbreak and prepare for the next one. WHO is also contributing to this effort through its R&D Blueprint.[3]
